# Novel *KISS1* Gene Mutation Leading to Male Hypogonadotropic Hypogonadism

**DOI:** 10.1055/a-2787-6622

**Published:** 2026-03-05

**Authors:** Leonie Wittner, Santosh Mahindrakar, Ali Yasin, Sandra Nicole Scheel, Wolfgang Hoeppner, Joachim Feldkamp

**Affiliations:** 1 9167 Medical School and University Medical Center East Westphalia-Lippe, Bielefeld University, Bielefeld, Germany; 2 91788 Academic Department of General Internal Medicine, Endocrinology and Diabetes, Infectiology, Klinikum Bielefeld Mitte, Bielefeld, Germany; 3 Bioglobe GmbH, Hamburg, Germany

**Keywords:** hypothalamic-pituitary-gonadal axis, puberty, infertility, reproduction

## Abstract

The human *KISS1* gene encodes the hypothalamic Kisspeptin, which is released in a pulsatile manner and binds the KISS1 receptor, that is located on gonadotropin releasing hormone neurons. This interaction ensures pulsatile gonadotropin releasing hormone secretion leading to induction of the hypothalamic-pituitary-gonadal axis and by this controls puberty onset. Disruption of this process is associated with hypogonadotropic hypogonadism. We identified a novel heterozygous *KISS1* variant c.-7C>T in two brothers diagnosed with hypogonadotropic hypogonadism. The mutation affects the Kozak consensus sequence of the *KISS1* gene and potentially interferes with *KISS1* gene expression. Consequently, this affects the hypothalamic-pituitary-gonadal axis resulting in hypogonadotropic hypogonadism. In both patients, complete development of primary and secondary male sex characteristics and stabilization of serum sex steroid hormone levels was achieved by testosterone therapy. Additionally, human chorionic gonadotropin and follicle stimulating hormone combination therapy in the older brother (patient 1) induced spermatogenesis and enabled fatherhood. Apart from this, we identified the heterozygous *CHD7* variant c.2690G>A in the younger brother (patient 2). However, the contribution of this variant to the pathogenesis of hypogonadotropic hypogonadism remains elusive.

## Introduction

Human reproduction is tightly linked to the hypothalamic-pituitary axis 1 . Gonadotropin releasing hormone (GnRH) neurons exhibit pulsatile activity and are key drivers for the induction of puberty and human reproduction 2 3 . GnRH deficiency is an oligogenic disease and exhibits a heterogeneous clinical presentation 4 5 . It can result in hypogonadotropic hypogonadism (HH), which is characterized by low gonadotropins and sex steroid hormone levels. Ultimately, HH prevents puberty onset and is associated with infertility 5 .

Pulsatile Kisspeptin 1 (Kp) activity induces GnRH secretion that activates the hypothalamic-pituitary-gonadal axis 6 . Interfered Kp signaling is associated with HH and lack of puberty onset 7 8 . Loss of function mutations of the human *KISS1* and *KISS1R* (KISS1 receptor) genes have been reported to cause normosmic and anosmic GnRH deficiency 7 8 , which frequently manifested in HH 9 10 11 12 13 14 15 16 .

Here, we report a novel *KISS1* mutation in two brothers leading to hypogonadotropic hypogonadism.

## Materials and methods

### Patient consent

Written informed consent was obtained from both patients.

### Next generation sequencing

DNA of both patients was isolated from whole blood samples using the QIAamp DNA blood Mini Kit (Qiagen, Hilden, Germany) according to manufacturer’s guidelines and next generation sequencing (NGS) was performed. Further clinically relevant deviations from the reference sequence apart from *KISS1* and *CHD7* gene mutations, were excluded with>98% probability.

### Multiplex ligation-dependent probe amplification

Deletions and duplications of the *ANOS1* , *FGFR1* , *PROK2* , *PROKR2, CHD7* , *GNRH1, GNRHR* , and *KISS1R* genes were analyzed via Multiplex ligation-dependent probe amplification (MLPA). DNA was isolated as described above. MLPA was performed using SALSA MLPA Kit P050-B2 CAH (MRC-Holland) according to manufacturer’s guidelines.

## Results

### Clinical presentation and management

Patient 1 is the older brother and was diagnosed at the age of 29 with idiopathic HH (normoosmic). HH was pronounced with luteinizing hormone (LH) serum levels below the detection limit and testosterone serum levels below the reference values. In line with this, he presented with absent development of male sex characteristics and infertility. Testosterone therapy successfully initiated puberty resulting in complete development of primary and secondary male sex characteristics and stabilization of serum sex steroid hormone levels. Later, he received a combination therapy of human chorionic gonadotropin (hCG) and FSH which induced spermatogenesis. In response to that, his spermiogram corresponded to the normal range regarding volume, time to liquefication, colour, total sperm count, concentration, consistence, pH, and motility. However, sperm morphology presented with a high proportion of abnormal heads and midpieces (teratozoospermia) ( Table 1 ). Ultrasound examination of the testes showed normal tissue and volume with a tendency towards the lower limit range (right 9.9 ml, left 10.9 ml). Patient 1 fathered two children.

**Table:** **Table 1** Spermiogram of patient 1 after hCG and FSH combination therapy.

Parameter	Result	Reference
Volume ejaculated	5.4 ml	> 1.5 ml
Time to liquefication	10 min	< 30 min
Colour	Milky-white	Milky-white
Total sperm count	313.2 mil	> 39 mil
Concentration	58 mil/ml	> 15 mil/ml
Consistence	Normal	Fluid
pH	8.5	> 7.2
**Motility (%)**	**Mean**	**Reference**
Rapid progressive	0	> 32
Slowly progressive	72.7	
Local progressive	2.3	-
Immotile	25	-
**Morphology (%)**	**Mean**	**Reference**
Normal	2.8	> 4
Abnormal heads	83	-
Abnormal midpieces	14.2	-
Abnormal principal pieces	0	-

Reference according to WHO laboratory manual for the Examination and processing of human semen FIFTH EDITION 2010.

Patient 2 is the younger brother. He presented at age 32 with a prepubescent appearance and was diagnosed with idiopathic HH: height 181 cm, arm span 190 cm, weight 90.8 kg, high voice, lack of male facial and body hair, sparse pubic hair (Tanner stage II), childlike penis (Tanner stage II). Ultrasound examination of the testes demonstrated low volume (right 2 ml, left 1.5 ml). The left testicle is palpable in the inguinal canal. X-ray examination of the left hand determined a bone age of 18 years. Hormone serum concentrations were below or at the lower reference limit ( Table 2 ). Patient 2 was also diagnosed with idiopathic HH (normoosmic). Likewise, testosterone therapy successfully induced complete development of primary and secondary male sex characteristics and stabilization of serum sex steroid hormone levels ( Table 2 ).

**Table:** **Table 2** Hormone serum concentration of patient 2 undergoing testosterone therapy.

Parameter	Hormone serum concentration			Reference
	before testosterone therapy	3 months after therapy initiation	10 months after therapy initiation	
FSH	1.1 mU/ml	< 0.5 mU/ml	< 0.5 mU/ml	0.7–11.1 mU/ml
LH	0.4 mU/ml	0.4 mU/ml	< 0.4 mU/ml	0.8–7.6 mU/ml
Estradiol	< 20 pg/ml	31.2 pg/ml	29.4 pg/ml	< 56 pg/ml
Testosterone	< 20 ng/dl	384 ng/dl	390 ng/dl	160–726 ng/dl
Prolactin	3.7 ng/ml	15.5 ng/ml	n. a.	2.5–17 ng/ml

The two patients came from Iraq to Germany. Here, they received medical treatment for their symptoms for the first time, whereupon they were diagnosed with HH. For this reason, the disease was diagnosed delayed.

**Figure 1:**
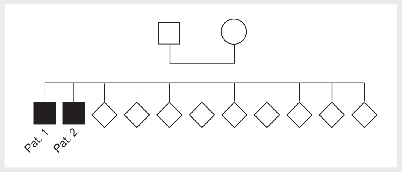
Family tree of patient 1 and 2. Patient 1 and 2 are the only siblings affected by idiopathic HH in their family.

Further siblings of patient 1 and 2 were not affected by idiopathic HH ( Figure 1 ).

### Identification of genetic aberrations

**Figure 2:**
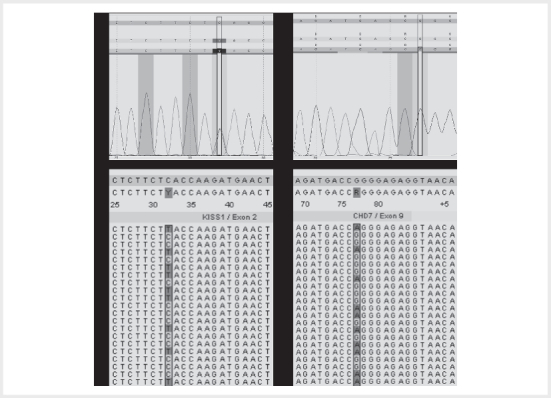
Identification of KISS1 variant c.-7C>T and of CHD7 variant c.2690G>A. **a** Sequence analysis of patient 1 and **b** sequence analysis of patient 2 indicate the heterozygous substitution C> T at position -7 of the *KISS1* gene. **c** Sequence analysis of patient 1 revealed no genetic aberration in *CHD7* . **d** Sequence analysis of patient 2 indicates the heterozygous substitution G> A at position 2690.

Due to the increased familial prevalence of idiopathic HH, we screened patient 1 and 2 for genetic aberrations associated with infertility. Whole genome analysis revealed the familial heterozygous *KISS1* variant c.-7C>T (dbSNP: rs369561225, frequency: A= 0.00071 (30/42216, ExAC)) in both patients ( Figure 2 a, b ). The mutation affects the mRNA 5’-untranslated region (UTR), where it is localized in the Kozak consensus sequence. It has been described earlier in a patient with Kallmann syndrome 17 .

Furthermore, only patient 2 exhibits the heterozygous *CHD7* (Chromodomain Helicase DNA Binding Protein 7) variant c.2690G>A, p.Arg897Gln (dbSNP: rs773685788, frequency: A= 0.00002 (2/83202, ExAC)) ( Figure 2 c, d ). Both chromosomes are affected by the gene change (transposition), indicating that it did not occur *de novo* . The variant has not been described earlier. However, the *CHD7* variant c.2690G>C at the same position has been demonstrated to cause HH 18 . PolyPhen-2 19 predicts the amino acid exchange p.Arg897Gln as “benign” with a score of 0.053 (sensitivity: 0.93; specificity: 0.64) in the HumVar model.

MLPA analysis of the infertility associated genes *ANOS1* , *FGFR1* , *PROK2* , *PROKR2, CHD7* , *GNRH1, GNRHR* , and *KISS1R* excluded large duplications and deletions.

## Discussion and conclusions

We identified the potentially disease causing familial heterozygous *KISS1* variant c.-7C>T in two brothers diagnosed with HH. Since the mutation occurred in two brothers, we assume that it was inherited. The single nucleotide polymorphism (SNP) at position -7 is located in the 5’-UTR within the Kozak consensus sequence, which is a eukaryotic ribosome binding site in close proximity to the translational start codon 20 . Therefore, the mutation potentially interferes with proper ribosome binding and translation of the Kp protein. Kp deficiency is associated with disturbance of the hypothalamic-pituitary-gonadal axis which can result in HH 7 8 .

Interestingly, the *KISS1* variant c.-7C>T has already been described in a patient with Kallmann syndrome 17 , which is a form of congenital HH that arises due to a lack of functional hypothalamic GnRH neurons and is characterized by deficiency of GnRH and sex steroid hormones as well as hypo- or anosmia 21 . Hypo- or anosmia occurs due to disturbance of the olfactory bulb development during embryogenesis. This results in aberrant migration of hypothalamic GnRH neurons, which originate in the medial olfactory placode 22 23 . A variety of gene mutations, including *KISS1* mutations, are associated with the pathogenesis of Kallmann syndrome 17 24 25 26 . However, isolated Kp deficiency in patient 1 and 2 presumably does not affect embryonic olfactory bulb development and GnRH neuron migration, explaining why both patients have a normal sense of smell.

Since both patients presented with similar symptoms, it is not clear to what extent the *CHD7* variant c.2690G>A identified in patient 2 contributed to the pathogenesis of HH. PolyPhen-2 classifies the mutation as benign. Nevertheless, this does not allow a prediction of the phenotype. The previously described *CHD7* variant c.2690G>C affects the same position and has been shown to cause HH 18 . Therefore, the *CHD7* mutation in patient 2 might have enhanced the pathogenesis of HH.

In conclusion, identification of the heterozygous *KISS1* mutation c.-7C>T added another genetic variant to the list of HH causing genome aberrations 24 , that will facilitate to identify the cause of male idiopathic HH. The role of the *CHD7* variant c.2690G>A in the pathogenesis of HH remains unclear.
